# Dependability Modeling and Assessment in UML-Based Software Development

**DOI:** 10.1100/2012/614635

**Published:** 2012-09-03

**Authors:** Simona Bernardi, José Merseguer, Dorina C. Petriu

**Affiliations:** ^1^Centro Universitario de la Defensa, Academia General Militar, Zaragoza, Spain; ^2^Departamento de Informática e Ingeniería de Sistemas, Universidad de Zaragoza, 50018 Zaragoza, Spain; ^3^Department of Systems and Computer Engineering, Carleton University, Ottawa, ON, Canada K1S 5B6

## Abstract

Assessment of software nonfunctional properties (NFP) is an important problem in software development. In the context of model-driven development, an emerging approach for the analysis of different NFPs consists of the following steps: (a) to extend the software models with annotations describing the NFP of interest; (b) to transform automatically the annotated software model to the formalism chosen for NFP analysis; (c) to analyze the formal model using existing solvers; (d) to assess the software based on the results and give feedback to designers. Such a modeling→analysis→assessment approach can be applied to any software modeling language, be it general purpose or domain specific. In this paper, we focus on UML-based development and on the dependability NFP, which encompasses reliability, availability, safety, integrity, and maintainability. The paper presents the profile used to extend UML with dependability information, the model transformation to generate a DSPN formal model, and the assessment of the system properties based on the DSPN results.

## 1. Introduction

Model-driven development [1] (MDD) is an evolutionary step that changes the focus of software development from code to models, with the purpose of automating the code generation from models. MDD emphasis on models facilitates also the analysis of nonfunctional properties (NFP) (such as performance, scalability, reliability, security, safety, or usability) of the software under development based on its models. These NFPs are finally responsible for the required quality of the software [2]. Among them, we address in this paper the dependability NFP. Dependability encompasses availability, reliability, safety, integrity, and maintainability as proposed in [3].

**Figure 1:**
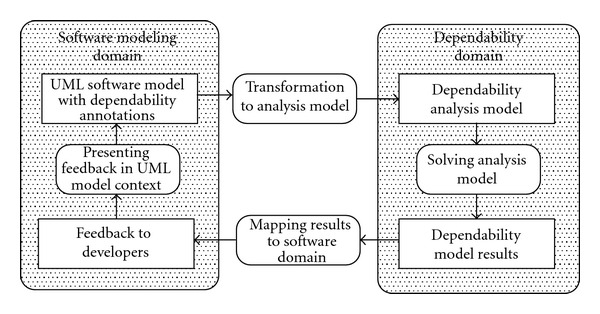
Integrating dependability modeling and analysis in a UML-based software development.

Many formalisms and tools for NFP analysis have been developed over the years. For example, queueing networks [4], stochastic Petri nets [5], stochastic process algebras [6], fault trees [7], or probabilistic timed automata [8]. One of the MDD research challenges is to bridge the gap between software models and dependability analysis models. An emerging approach for the analysis of different NFPs, dependability included, is given in Figure 1. It consists of the following steps: (a) to extend the software models used for development with annotations describing dependability properties; (b) to transform automatically the annotated software model to the formalism chosen for dependability analysis; (c) to analyze the formal model using existing solvers; (d) to assess the software based on the results and give feedback to designers. Such a modeling→analysis→assessment approach can be applied to any software modeling language, be it general purpose such as the Unified Modelling Language [9] (UML), or domain specific such as AADL [10] or SysML [11].

In the case of UML-based software development, the extensions required for NFP-specific annotations are defined as UML profiles [9], which provide the additional advantage of being processed by standard UML tools without any change in the tool support. OMG adopted the MARTE [12] profile (see Appendix A), which extends UML for the real-time domain, including support for the specification of schedulability and performance NFPs. We use the dependability modeling and analysis [13] (DAM) profile (see Appendix A) to extend the UML models with dependability concepts and then transform the extended UML model into a Deterministic and Stochastic Petri Net (DSPN) model (see Appendix B). The results of the DSPN model are converted to the software domain and are used to assess system dependability measures.

The work [14] formalized the methodology in Figure 1. In this paper, we rigorously apply this formalization, through a case study, in the context of UML-based development.  Section 3 accomplishes the modeling step of the methodology.  Section 4 applies the transformation step.  Section 5 focusses on the analysis step.  Section 6 explores the assessment step.

## 2. Case Study: The Voter

According to Avižienis et al. [3], the means developed to attain system dependability in the past 50 years can be grouped into four categories: fault tolerance, fault prevention, fault removal, and fault forecasting. The case we present pertains to the fault tolerance field, which aims to improve dependability by avoiding service failures in the presence of faults.

Fault tolerance [15] provides different well-known techniques mainly based on error detection and system recovery. Voting as well as software and hardware replication are the techniques we use here. Concretely, we present a voter mechanism whose purpose is to mask faults arising in computations carried out with data acquired by a* sensor*.

We are considering a* sensor* which monitors (a part of) a generic plant, such as an industrial automation system. The* sensor* periodically sends raw collected data to an *application* that carries out a heavy and critical computation with it. We replicate the computation through different nodes with the purpose of increasing the fault tolerance of the* application*. However, it can happen that one or more of the* replicas* are affected by faults, that is, they do not complete their computations as scheduled, may be due to a node failure, a memory leak or another software bug. Our system deals with this situation by implementing a voting mechanism to mask one fault, that is, the system provides results despite the presence of a fault.

Voting algorithms are often used along with recovery mechanisms, which bring back the system to a healthy state when the voting cannot be accomplished, that is, when the faults cannot be masked. For the sake of simplicity, we will not consider recovery strategies in this example.

We propose an initial UML design of the voter containing a deployment diagram and a set of state machines (UML-SMs). The design model illustrates the following:

- how dependability techniques can be modeled with UML behavioral diagrams and DAM annotations introduce dependability parameters;
- how DAM leverages this design for dependability analysis purposes.

**Figure 2:**
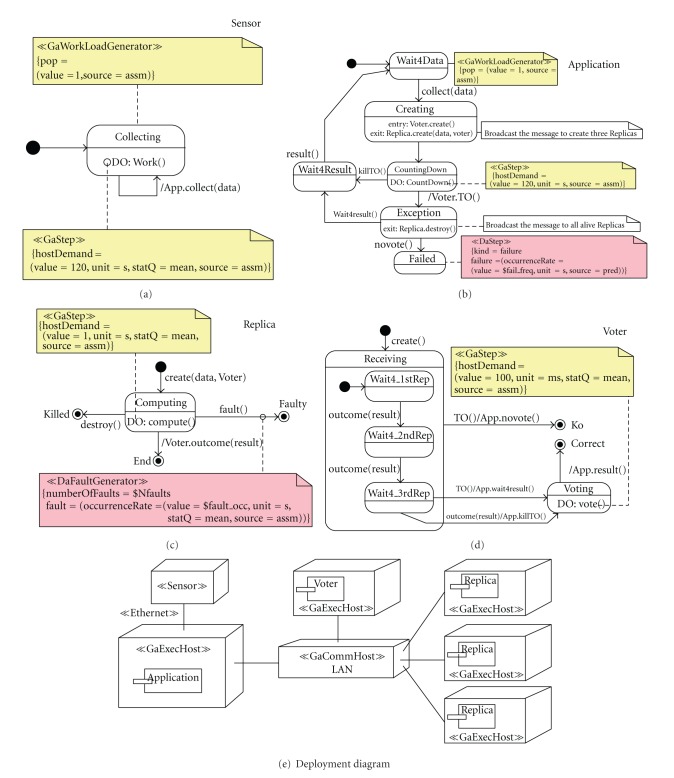
UML design of the voter.

The deployment diagram, Figure 2(e), depicts the hardware nodes in which the identified software components (sensor, application, and replicas) execute and also the communication networks linking them. We consider a fully distributed system architecture to increase dependability. In fact, the distribution of the components is a principle in dependability modeling.

The voter exhibits a discrete behavior for which UML-SMs are well suited. According to the UML interpretation, a SM specifies the behavioral pattern for the objects populating a class, as in the case of the UML-SM for the three voting replicas (Figure 1(c)). Alternatively, a UML-SM can also specify the behavior of a software component, such as the application, voter or sensor embedded components (Figure 1(a, b, d)).

## 3. Dependability Modeling

UML-SMs are widely used to pragmatically model the “correct” behavior of a system, that is the behavior in absence of faults. However, dependability modeling demands to specify also the system behavior under different* fault assumptions*, and to characterize the * system failures*. Furthermore, in case of repairable systems, the * repair and reconfiguration activities* that remove basic or derived failures from the system need to be modeled. In order to define the system fault assumptions, a software engineer has to consider the following main issues:

1. which components can be affected by faults and in which states,
2. the maximum number of faults that can concurrently affect the system components,
3. the complete fault characterization, such as the fault occurrence rate.

Failure characterization consists in determining the failure modes and, in particular, the system failure states.

UML does not provide sufficient capabilities for a complete and rigorous modeling of all the aforementioned dependability concerns. However, the DAM profile augments a UML design with annotations that target the dependability specification. Being constructed as a specialization of MARTE, DAM ensures compatibility with the UML diagrams. The MARTE part of interest to DAM is the one devoted to quantitative analysis, also known as GQAM (see Appendix A). In fact, DAM specializes GQAM, creating a framework for the specification and analysis of dependability.

### 3.1. State Machines Specification

 Our UML-SMs specification illustrates how the engineer can model specific dependability techniques while describing the system normal behavior. Concretely, we have leveraged the UML-SMs to propose a design for the* voting* mechanism and computation by a *replica*.

Following the UML-SM of the application in Figure 2(b), we see that it collects the data from the sensor then, it creates the voter and three replica processes (see state C reating) and starts a countdown. The CountingDown state discerns between the correct behavior of the system and masking or abnormal behaviours. It is considered that the system behaves correctly if the replicas can normally carry out their computations before the CountDown() completes. Then, the application eventually receives from the voter the killTO() event and the r esult() of the computation. The* fault masking* behavior occurs, instead when the time out expires before the voter can kill it; the application informs the voter by sending the TO() event. The application enters in Exception state, but it can still receive the wait4result() event and later the result(), which has been produced by the voter based on the outcome() of the remaining two no-faulty replicas. Please note in the UML-SM of the voter that if the TO() event is received after the outcome of the second replica, then the *voting* is still performed, so one faulty replica can be tolerated. Finally, the system abnormal behavior occurs when no vote is produced and the voter notifies it the application, which enters in Failed state.

### 3.2. DAM Specification

 The* fault masking* specification (i.e.,* voting* and* replica* computation) has been modeled by using UML-SMs, however the* fault assumptions* and the* system failures* still need to be specified. To achieve this, DAM provides a small yet sufficient set of extensions, (i.e., stereotypes and tagged values) which are DaStep, DaComponent and DaService. 

The DaStep stereotype is meant to be applied to basic computational steps, which in the context of SMs are mostly states and transitions. It allows a complete specification of * failures* or * hazards* (for safety-related systems),* errors*, and* recovery actions*. In our example, we have defined a* failure state* in the application UML-SM, which corresponds to the system failure. It is worth to note that this is a simple case since, in general, a system can be subject to different failure modes and each failure is a combination of the system component failures. DAM also supports the failure specification in the general case.

The tag failure provides attributes to thoroughly describe a software failure, such as the failure occurrence rate (as shown in the example), but also the mean time to failure (MTTF), mean time between failures (MTBF), domain and detectability of failure, and logical condition that leads to failure. Concerning* errors*, DAM allows one to specify the error latency and probability, while for* recovery actions* one can specify the rate, duration, distribution, and coverage factor.

DaComponent and DaService, although not illustrated in our example, are of primary importance for the dependability specification. The former describes aspects such as availability, reliability, faults, failures, or errors affecting the software components; while the latter specifies the same aspects but in the context of software services. *Repair and reconfiguration activities* are specified through the DaRepair and DaRecovery stereotypes.

Another aspect to be considered is the definition of the fault events, which can be represented as a special type of workload. The stereotype DaFaultGenerator provides the means to model a fault injector. In the example, we assume that only replicas can be affected by faults, so we apply this stereotype to the SM transition that leads a replica to a faulty state. The tag NumberOfFaults is set to an input variable, $*Nfaults*, for sensitivity analysis purposes. The tag fault allows to completely specify the* fault assumption* within DAM, concretely its occurrence rate, latency, occurrence probability, occurrence distribution, persistency, and duration.

Finally, the definition of* dependability measures* during this stage of the design is of primary importance for the engineer to clearly specify the goals of the analysis. In this case, we have used the failure description in the DaStep to define the measure of interest as the inverse of Mean Time To Failure (MTTF), which represents the application failure occurrence rate. DAM allows one to specify a wide variety of measures, such as the Mean Time Between Failures or the availability.

### 3.3. MARTE Specification

 A DAM specification is useful for addressing most of the quantitative and qualitative dependability aspects. However, for analysis purposes we may need to enhance the specification with some quantitative parameters provided by MARTE annotations. For instance, we need to indicate the population of the system and the duration of the involved activities. We use a subset of GQAM stereotypes to specify: (1) the number of objects populating a UML-SM (as GaWorkloadGenerator stereotype with pop tag), (2) the timing duration of the UML-SM activities (as GaStep stereotype with hostDemand tag), and (3) the type of DD resource for informative purposes.

In Figure 2 we defined an initial population only for the application and the sensor, while the other objects (i.e., the replicas and the voter) are dynamically created. The timing durations of the UML-SMs activities have different statistical meaning. For example, the duration of the TimeOut of the application is a constant value, while the duration of the compute activity performed by the replicas is a mean value. Concerning the type of hardware resource, we identify, through the GaExecHost stereotype, the processors where the UML-SM activities execute. The communication nodes are stereotyped as GaCommHost stereotype, see also Figure 2.

## 4. Transformation to Formal Model

The UML-DAM design of the voter specifies both the behavioral and dependability properties. The next challenge for the engineer is to analyse the system dependability before the implementation phase. The proposed approach is to transform the UML-DAM specification into a formal model appropriate for dependability analysis. In our case, DSPN (Deterministic and Stochastic Petri Net, see Appendix B) is such a formal model, which can be automatically generated from the UML-DAM design. In order to derive the DSPN model representing the whole system, we propose to start by transforming each UML-SM into a * component* DSPN following two steps.

1. Derive the structure of a *component* DSPN from a UML-SM.
2. Derive the fault assumptions, timing specification, and the initial marking of the DSPN from the MARTE-DAM annotations associated with the respective UML-SM.

After deriving all *component* DSPNs, they are composed according to the SMs interactions.

### 4.1. Deriving the DSPN's Structure

 The model transformation is based on predefined patterns: for each SM model element, we derive a DSPN subnet with labeled places and transitions. The labels have a double purpose: (a) to compose the DSPN subnets by merging the places or transitions with the same label and (b) to enable the tracing of the SM-to-DSPN mapping, an important feature needed to support the feedback of analysis results to the original SM.

In the following, we succinctly describe the transformation of the most important model elements of a SM (states, events and transitions), to allow the reader to grasp the general idea of the approach. Note that the SM-to-DSPN transformation has been completely automated (see Appendix C).

#### 4.1.1. States

**Figure 3:**
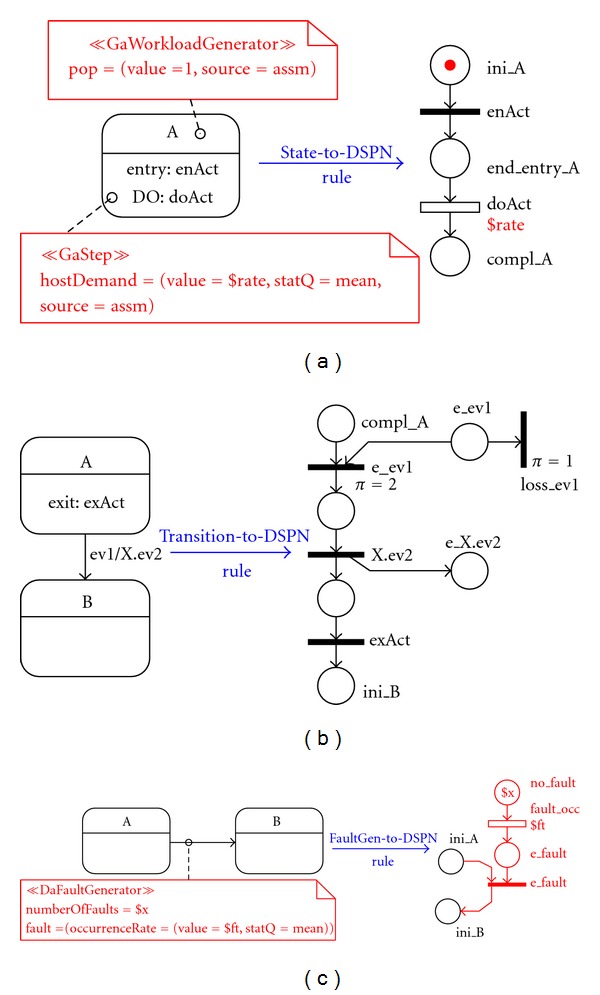
SM-to-DSPN patterns.

Figure 3(a) shows the transformation of a simple state with an entry action and a do-activity. The DSPN subnet contains two causally connected transitions: one immediate and one timed that model, respectively, the entry action and the do-activity. The compl _ A place, when marked, represents the state reached by the SM once the do-activity has been completed. 

#### 4.1.2. Events

 Events are mapped onto DSPN places, labeled as e_eventname; they represent *mailboxes* whose marking indicates the number of event occurrences of the same type.

#### 4.1.3. Transitions

 The transformation pattern of a transition * event/action* is shown in Figure 3(b). The transition fires when the* event* occurs (i.e., the DSPN place e_ev1 becomes marked), but only if the SM is in the source state of the transition (i.e., comp_A is marked), otherwise the event is lost. The consumption and the loss of an event are modeled by two immediate DSPN transitions: e_ev1 and loss_ev1, respectively, with the * event mailbox* place e_ev1 as input place. The former has greater priority (*π* = 2) than the latter (*π* = 1), so that when a conflict arises the former eventually fires. Then, the execution of the action * X.ev2* generates an event* ev2* for a SM named * X*. Finally, the exit action* exAct* in state * A* is carried out and the state* B* represented by the place ini_B is reached.

### 4.2. Setting the DSPN Parameters

 MARTE-DAM annotations in a SM are mainly transformed to parameters of the * component* DSPNs. In general, the tagged values specified as assumed values (*source=assm*) are mapped to DSPN input parameters, while predicted values (*source=pred*) to output dependability measures.

#### 4.2.1. Fault Assumptions

The fault generator (*DaFaultGenerator* stereotype) is translated into a new DSPN subnet shown in Figure 3(c), which generates fault events. In the voter, the fault generator DSPN subnet generates fault events for the replicas. The *value* field of the *numberOfFaults* tagged-value is a variable that is translated to the initial marking of the DSPN subnet, which specifies the maximum number of faults that may occur in the SM. The *value* field of the *occurrenceRate* tagged-value is also a variable used to define the mean firing time parameter of the transition whose firing represents a fault occurrence.

#### 4.2.2. Timing Specs

 The * doActivities* annotated with corresponding processing demands, are translated into timed transitions, as in Figure 3(a), and the *value* field of the *hostDemand* tagged-value is mapped to the input time parameter of the corresponding DSPN transition. When the mean statistical qualifier (statQ=mean) is associated to a tagged value, the DSPN parameter represents the mean value of an exponentially distributed random variable, otherwise it models a deterministic value. In real-time system models it is useful to assume that some of the computing times are stochastic (e.g., the voting time in the voter) while others deterministic (e.g., the time-out in the application); both are naturally modeled in the DSPN formalism.

#### 4.2.3. Initial Marking

The initial population of a SM, tagged-value *pop*, is translated into the initial marking of the corresponding *component* DSPN, as seen in Figure 3(a). In the voter, only the sensor and the application are characterized by an initial population, each represented by the initial marking of the corresponding * component* DSPNs.

## 5. How to Approach the Analysis

In the UML design, the software engineer specifies the output dependability measures of interest using the DAM profile. Dependability analysis consists in computing such measures by solving the DSPN model; the results will be eventually interpreted in the application domain and used for system assessment. In the voter example, the measure used to assess the fault-tolerance of the system is the failure occurrence rate of the *application*, see Figure 2(b).

The proposed analysis is carried out through the following tasks: (1) derive the dependability DSPN model of the whole system, (2) define the dependability measures at DSPN level, and (3) choose and run the appropriate DSPN solver.

### 5.1. Derivation of the Dependability DSPN Model

**Figure 4:**
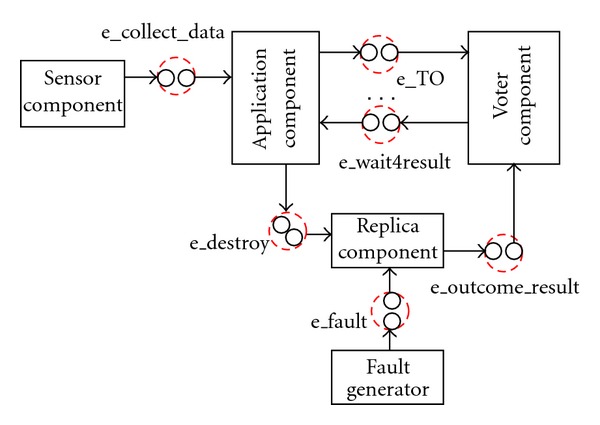
Composition of the DSPN *subnets* over interface places.

 The DSPN dependability model of the entire system is automatically constructed (see Appendix C) by composing the * component* DSPN subnets derived from the UML-SMs by model transformations (see Section 4). More specifically, the composition takes place by merging the interface places with matching labels that belong to different *component* DSPNs, as illustrated in Figure 4. There is a pair of interface places with matching labels e_eventname for each event generated/consumed by the SMs: one place belongs to the *component* representing the sender SM and the other to the *component* representing the receiver SM. The DSPN composition replaces the pair of interface places by a single place (bigger dotted circle).

### 5.2. Definition of the DSPN Dependability Measures

**Figure 5:**
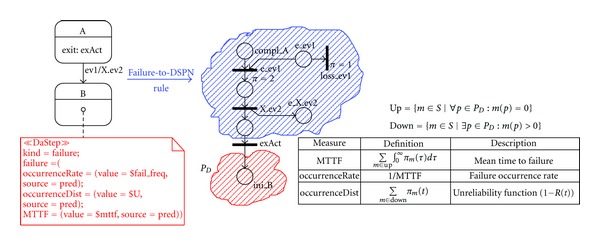
Definition of DSPN dependability measures.

 The DAM values, specified with the Value Specification Language [12] (VSL, see Appendix A) as predicted values (source=pred), correspond to output dependability measures to be computed by solving the DSPN model. A DSPN dependability measure is a stochastic measure defined over the set *S* of DSPN markings (i.e. states) reachable from the initial marking (see Appendix B). In the composed DSPN model, the state set *S* can be partitioned into two subsets containing the states when the system operates normally (Up) and the failure states (Down), respectively. The main concern in the definition of a DSPN dependability measure is the identification of the system failure states considering the DAM predicted values.  Figure 5 (center) shows the portion of net obtained from the transformation of the UML SM on the left, according to the patterns defined in Figure 3. The red cloud contains the place corresponding to the SM state *B*, specified with DAM as a failure state, while the blue cloud includes the rest of the DSPN places. Observe that, in general, there can be several places in the red cloud, depending on the number of SM states specified as failure states; let's denote such a set of places as *P*_*D*_. Then, the set of failure states corresponds to the set of DSPN markings Down where at least a place in *P*_*D*_ is marked. Conversely, the set of operational system states corresponds to the set of DSPN markings Up where none of the places is marked.

The table in Figure 5 (bottom-right) shows the definition of some common DSPN reliability measures, that can be mapped from the homonym DAM tags attached to the state *B* on the left, where *π*_*m*_(*t*) is the probability of being in a given marking *m* ∈ *S* at a given instant *t* ≥ 0. The definition and computation of such formulas are commonly supported by DSPN tools currently available in the Petri Net community (see Appendix C) (Similar formulas apply to compute availability measures).

In the example, the measure to be predicted is the failure occurrence rate of the control *application* (see DAM annotation in the SM of the* application* in Figure 2).

### 5.3. Choice of the DSPN Solver

 Once the dependability metrics of interest, specified in the UML design with DAM, have been mapped onto the corresponding metrics at DSPN level, we are ready to solve the composed DSPN model to get estimated values of such metrics. The choice of the appropriate DSPN solver depends mainly on two factors: (a) the characteristics of the DSPN model and (b) the dependability metric to be evaluated. Concerning the first factor, numerical methods derive a system of linear equations from the DSPN model and solve it by using either exact or approximate mathematical techniques [16]. Unfortunately, existing DSPN analytical methods suffer from the well-known state-space explosion problem. Discrete event simulation can be used as an alternative method [17].

The second factor affects the type of analysis to be used: transient versus steady state. For transient analysis, the system behavior is observed during a finite time interval, while for steady state analysis system behavior is observed for a *sufficiently large* period so that the analysis becomes time-independent. Typically, the reliability (survival) function is computed under transient state assumption while mean value metrics, such as MTTF, can be estimated in steady state.

## 6. Analysis and Assessment Results

Let us consider the following question: could the system carry out its computations in the presence of faults due, for example, to software bugs or nodes failures? In order to answer such a question, the engineer should assess the proposed system design both with and without the fault-masking mechanism. In the first case, the UML design is as shown in Figure 2, while in the second case the voter is omitted and only one replica is created. Two different DSPN models will be derived automatically, and the failure occurrence rate will be computed for each one.

We carried out sensitivity analysis under the steady state assumption, using the simulator implemented in the GreatSPN tool (see Appendix C) to solve the two DSPN models. Two fault input parameters were considered: the replica fault rate and the maximum number of faults that may affect the replicas during the experiment.

**Figure 6:**
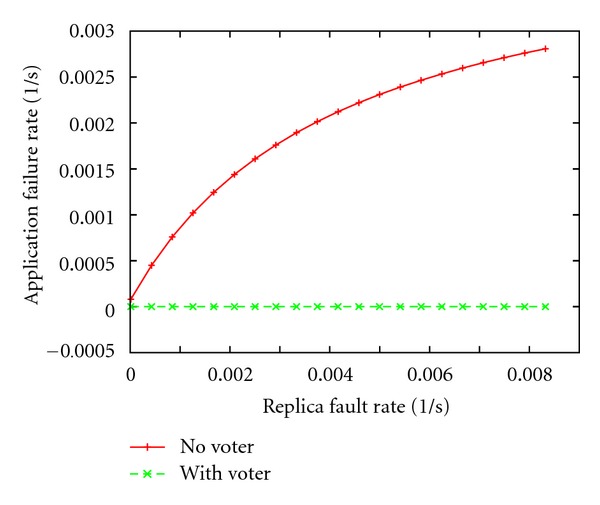
*Application* failure rate (fail/sec.) versus the replica fault rate under one fault-assumption.

**Figure 7:**
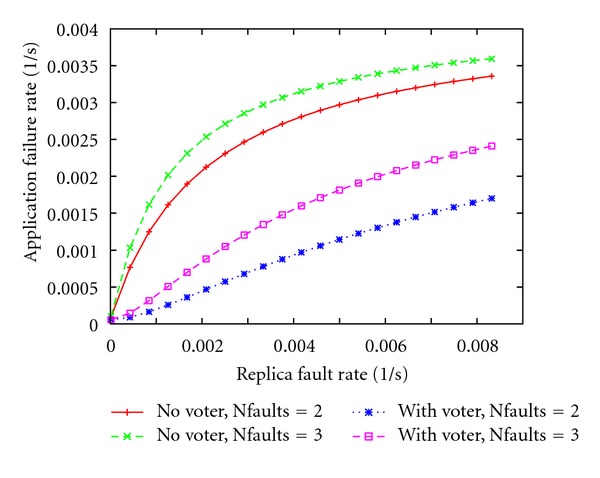
*Application* failure rate (fail/sec.) versus the replica fault rate under multiple fault-assumption.

 Figures 6 and 7 show the results of the analysis, where the* application* failure rate is plotted versus the* replica* fault rate. In both figures, the cases with and without the voter are represented.  Figure 6 shows the measure under the *one-fault assumption*, that is, only one fault may occur during the experiment, so at most one replica is affected.

Figure 7 shows the results in the case when two or three independent faults may occur concurrently. Observe that in the first design (“with voter” case), multiple faults may affect different replicas. On the other hand in the second design (“no voter” case), where just one replica is present, considering two/three concurrent and independent faults is equivalent to assume, respectively, two/three times as much as the replica fault rate (*x*-axis).

As expected, the* application* failure rate increases as the number of fault occurrences grows from one to three. Moreover, when the replica fault rate grows, the probability that a replica fault affects the normal application behavior increases, and so does the application failure rate. On the other hand, when triple redundancy and voting is designed, the application is tolerant to a single replica fault, independently of the replica fault rate (Figure 6, green curve).

It is worth noting that the proposed analysis approach is flexible and powerful, especially due to the automation of the model transformation technique. The automatic derivation of DSPN models is flexible enough to easily manage different UML designs that specify different fault-tolerance solutions. Thus timely feedbacks can be provided to the software engineers when they need to assess dependability solutions for a given design.

## 7. Related Work

Paper [18] extensively surveys works on dependability modeling and analysis of software systems specified with UML. The survey analyses 43 papers from the literature published in the last decade on the topic. Herein, we consider the ones that mainly focus on reliability and availability analysis and propose model transformations which can be automated.

The most comprehensive approach has been proposed in [19, 20], where a UML profile for annotating software dependability properties compliant with the taxonomy and basic concepts from [3] is proposed. A model transformation process derives timed Petri net models via an intermediate model from the annotated UML models. The approach supports the specification of error propagation between components, as well as independent and dependent failures. In particular, it is possible to discriminate between normal and failure states and events, and to assign common failure mode occurrence tags to redundant structures. The main drawback of this work is the introduction of unnecessary redundant information in the UML model, as sometimes the joint use of more than one stereotype is needed.

Pai and Dugan [21] present a method to derive dynamic fault trees from UML system models. The method supports the modeling and analysis of sequence error propagations that lead to dependent failures, reconfiguration activities, and redundancies.

The papers [22–25] address specifically the reliability analysis of UML-based design. D'Ambrogio et al. [22] define a transformation of UML models into fault tree models to predict the reliability of component-based software. Cortellessa and Pompei [23] propose a UML annotation for the reliability analysis of component-based systems, within the frameworks of the SPT [26] and QoS&FT [27] profiles. The annotations defined in [23] are used by Grassi et al. [24, 25] where a model-driven transformation framework for the performance and reliability analysis of component-based systems is proposed. The method uses an intermediate model that acts as bridge between the annotated UML models and the analysis-oriented models. In particular, discrete time Markov process models can be derived for the computation of the service reliability.

Finally, the work [28] proposes a model-to-model transformation technique to support the availability evaluation of railway control systems. The availability model is a repairable fault tree that is automatically generated from the UML models (use case, component, and state machine diagrams), properly annotated with MARTE and DAM extensions.

## 8. Conclusion

 A standard specification framework is yet needed for dependability assessment of UML-based specifications. DAM is a step toward this goal, as it is a comprehensive approach attempting to unify a great number of efforts carried out by the researchers in the last decade.

Software quality includes a number of very different NFPs (e.g., security, performance, and dependability), which are often in conflict with each other [29]. In this context, the MARTE-DAM profile is a promising common framework for the specification of different NFPs in UML-based design. We envisage that a future research goal is to devise model transformation techniques that support a comprehensive analysis in presence of conflicting NFPs (e.g., performability, vulnerability, and survivability issues), in order to provide trade-off solutions to the software engineer. 

## Figures and Tables

**Figure 8:**
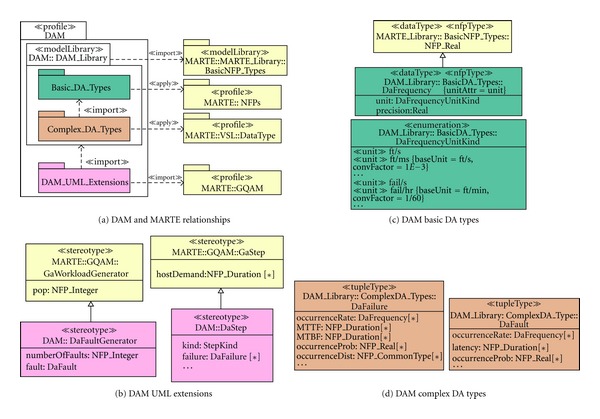
UML extensions for dependability modeling.

**Figure 9:**
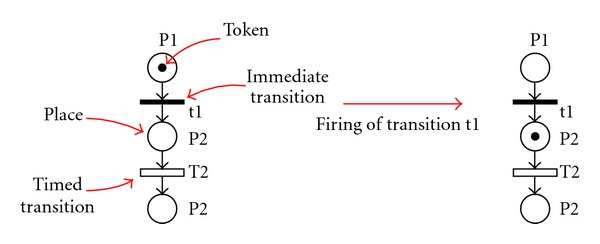
Petri net basic concepts.

## Appendix

### A. MARTE and DAM Profiles

#### A.1. MARTE

The “UML Profile for Modeling and Analysis of Real-Time and Embedded systems” [12] is an OMG standard profile that extends UML in a lightweight fashion (i.e. via the standard UML extension mechanism including stereotypes, tagged values, and constraints) and enables the specification of both quantitative and qualitative nonfunctional properties (NFP) in the form of annotations attached to UML model elements.

The Value Specification Language (VSL), which is a part of MARTE, provides the ability to express NFP types (defined in the so called MARTE library), values of NFP types, as well as variables, constants, and expressions. All of these are used by the modeler to assign values to tags, according to the VSL syntax (the annotations in Figure 2 show several examples). Tags of NFP types are characterized by several qualifiers:* source* defines the origin of the specification (such as required, assumed, predicted, and measured); statQ defines the type of a statistical measure (such as a maximum, minimum, or mean);* unit* indicates the measurement unit for a given NFP.

Beside VSL, another important feature of MARTE is a general analysis framework called the “General Quantitative Analysis Model” (GQAM) subprofile, which defines the foundation concepts common to different analysis domains. GQAM is specialized in MARTE to provide support for two kinds of analysis: schedulability (subprofile SAM) and performance (subprofile PAM).

#### A.2. DAM

The dependability analysis and modeling (DAM) profile specializes MARTE for dependability modeling and analysis, (Figure 8(a)). The entire set of DAM stereotypes, as well as the set of UML metaclasses extended by stereotypes can be found in [13]. A DAM subset supports the specification of system dependability properties at service level (e.g., a *DaService* use case) or at component level (e.g., a* DaComponent* class). Other stereotypes can be used to specify fault-tolerance redundancy structures (e.g., a *DaVariant* class). Finally, some stereotypes enable the characterization of the threats affecting the modelled system (e.g., a* DaFaultGenerator* event, a* DaStep* state) and the recovery strategies (e.g., a *DaReplacementStep* action).

According to UML, each DAM stereotype is made of a set of tags that define its attributes. For example, the * DaFaultGenerator* stereotype has* numberOfFaults* and* fault* as tags (see Figure 8(b)). The former indicates the number of concurrent faults and the latter characterizes the nature of the fault. DAM uses the MARTE library of basic NFP types for the definition of tag types and relies upon the MARTE VSL for the specification of tagged-values. It also defines new dependability specific types either as specialization of basic NFP types (e.g., the * DaFrequency* type in Figure 8(c) is a NFP real type, characterized by a fault/failure frequency unit), or as complex types, which consist of a set of basic NFP types (e.g., the *DaFault* and* DaFailure* in Figure 8(d)).

### B. Introduction to Petri Nets

A Petri net (PN)—shown in Figure 9—is a bipartite graph, in which the vertices can be either transitions or places. The transitions, graphically depicted by bars, represent events that may occur in the system; the places, represented by circles, are used to model conditions. The directed arcs, shown by arrows, describe which places are pre- or postconditions for which transitions. Places may contain tokens, depicted by black dots; the (initial) distribution of tokens over the places of a PN is called (initial) marking.

The PN dynamics is governed by the transition enabling and firing rules. A transition is enabled whenever there is at least a token in each of its precondition places, and it may fires if there are not enabled transitions with higher priority. When it fires, a token is consumed from each of its precondition places and a token is produced in each of its postcondition places (see Figure 9). A reachable marking is then a marking reached through the firing of a transition sequence from the initial one. In Deterministic and Stochastic Petri Nets (DSPNs), common metrics are the probabilities associated to the reachable markings, which can be either time-dependent (transient metrics) or time-independent (steady-state metrics).

DSPNs are characterized by two types of transitions: immediate and timed. Once enabled, an immediate transition fires immediately while a timed transition has an associated firing delay, which can be a constant value (deterministic) or a mean value of the negative exponential distribution (stochastic).

Place and transition labels have been introduced to enable the net composition. In particular, the places (transitions) belonging to two different net components and having the same label are merged into a unique place (transition), where the set of its input/output arcs is the union of the sets of the input/output arcs of the merged places (transitions).

### C. Tool Support

The automation of the modelling→analysis→assessment chain involves different tools. For the modelling step, several tools support UML design. Concretely ArgoSPE (http://argospe.tigris.org/), which is based on ArgoUML (http://argouml.tigris.org/), translates UML state machines into DSPNs automatically. ArgoSPE produces the DSPN in the format of GreatSPN (http://www.di.unito.it/~greatspn/index.html), a tool for the analysis and simulation of DSPN. Also TimeNET (http://www.tu-ilmenau.de/sse/timenet/) is a useful tool for the analysis of DSPNs. Regarding DAM profile, currently it has been implemented as a plug-in (http://webdiis.unizar.es/GISED/?q=tool/dam-profile/) for the Papyrus tool, but it has not been yet integrated with the analysis tools. MARTE has also been implemented for Papyrus and MagicDraw.
